# Fibronectin-Grafted Titanium Dental Implants: An* In Vivo* Study

**DOI:** 10.1155/2016/2414809

**Published:** 2016-06-06

**Authors:** Yu-Chi Chang, Kuo-Ning Ho, Sheng-Wei Feng, Haw-Ming Huang, Chia-Hsun Chang, Che-Tong Lin, Nai-Chia Teng, Yu Hwa Pan, Wei-Jen Chang

**Affiliations:** ^1^School of Dentistry, College of Oral Medicine, Taipei Medical University, Taipei 110, Taiwan; ^2^Graduate Institute of Biomedical Materials & Tissue Engineering, College of Oral Medicine, Taipei Medical University, Taipei 110, Taiwan; ^3^Department of International Logistics, Chung-Ang University, Seoul 156756, Republic of Korea; ^4^Dental Department, Taipei Medical University Hospital, Taipei 110, Taiwan; ^5^Department of General Dentistry, Chang Gung Memorial Hospital, Taipei 105, Taiwan; ^6^Graduate Institute of Dental & Craniofacial Science, Chang Gung University, Taoyuan 333, Taiwan; ^7^Dental Department, Taipei Medical University, Shuang-Ho Hospital, Taipei 235, Taiwan

## Abstract

Modification of the physiochemical properties of titanium surfaces using glow discharge plasma (GDP) and fibronectin coating has been shown to enhance the surface hydrophilicity, surface roughness, cell adhesion, migration, and proliferation. This* in vivo* study aimed to evaluate the bone integration efficacy of a biologically modified implant surface. Two different surface-modified implants (Ar-GDP and GDP-fib) were placed in the mandibular premolar area of six beagle dogs for 2–8 weeks. Three techniques [histologic evaluation, resonance frequency analysis (RFA), and microcomputed tomography (micro-CT) evaluation] were used to detect the implant stability and bone-implant contact. The implant stability quotient values of GDP-fib implants were significantly greater than the Ar-GDP implants at 2 and 4 weeks (*P* < 0.01). The bone volume/total volume ratio of GDP-fib implants was greater than the Ar-GDP implants in micro-CT evaluation. A high positive correlation was observed between RFA and micro-CT measurements. At 2 weeks, osteoblasts were seen to line the implant surface, and multinuclear osteoclasts could be seen on the surface of old parent bone. After 8 weeks, a majority of the space in the wound chamber appeared to be replaced by bone. Enhancement of the stability of biologically modified implants was proved by the results of RFA, micro-CT, and histological analysis. This enhanced stability may help fasten treatment and be clinically beneficial.

## 1. Introduction

In 1952, Per-Ingvar Brånemark et al. reported that titanium was biocompatible with bone and defined osseointegration as the direct structural and functional connection between ordered living bone and the titanium surface [1]. Osseointegration of dental implants depends on the molecular structure of the implant surface as well as cellular responses. It usually occurs during the surgery and throughout the healing process and is affected by several factors, such as bone quality and quantity, surgical techniques used [2–4], implant loading conditions [5], implant materials [6], implant surface characteristics [7, 8], and implant design.

Modification of the surface properties of implants can help improve cell attachment and promote bone healing. Previous studies have examined various surface treatment methods (e.g., laser treatment, blasting with abrasive particles [9], anodic oxidation [10], acid etching, and plasma spraying [11–16]) that modify the physicochemical properties of the implant surface and improve its contact with the bone. It has been shown that glow discharge plasma (GDP) technology can be used for surface sterilization and modification [17, 18], creation of biofunctional groups, and application of functional proteins on the titanium surfaces [19, 20]. In other words, it is useful for the creation of functional biointerphases and improvement of the biocompatibility of materials.

Previous studies have shown that coating the titanium surface with fibronectin, a protein crucial for cell growth, migration, and differentiation [21, 22], can help enhance the surface properties and cellular performance. Surface wettability and roughness are also higher with fibronectin coating compared to no coating [23]. The surfaces of GDP-fib titanium discs (0.400 *μ*m; 5.4 ± 0.8°) are significantly rougher and more hydrophilic than Ar-GDP specimens (0.170 *μ*m; 36.8 ± 8.5°). The fluorescein isothiocyanate (FITC) labeling ensures the formation of fibronectin coating. This grafted fibronectin usually exhibits spotty distribution instead of monolayer formation, and the number of fibronectin dots on the titanium surface increases positively with the concentration of fibronectin solution used. Enhancement of cell adhesion and differentiation on the fibronectin-linked titanium surface has also been observed [13, 24]. MTT ((3-4,5-dimethylthiazol-2-yl)-2,5-diphenyl tetrazolium bromide) assays and SEM (scanning electron microscope) images have demonstrated that the number of cells on Ar-GDP and GDP-fib specimens is greater than that on the original specimens after 24 hours of culture. Moreover, a morphological alternation of MG-63 cells from spindle to more stellar shapes with extensive filopodia that establish contact with each other as well as the substrate has been observed upon using the fibronectin-grafted titanium discs as substrate [24].

The use of GDP treatment for* in vivo* studies dates back to 1989 [25] when Carlsson et al. utilized an animal model consisting of rabbits to compare the removal torque and histology of GDP-treated implants and non-GDP-treated implants after 6 weeks* in situ*. They reported no qualitative or quantitative differences in this study. Conversely, MacDonald et al. found that radiofrequency plasma glow discharge pretreatments could promote osseointegration of Ti6Al4V rods in a rat model [26]. However, to the best of our knowledge, there are no other studies that have carried out an* in vivo* analysis of implants pretreated with GDP and/or fibronectin grafting.

Recent implant therapies tend to focus on the reduction of treatment time by immediate implantation, immediate loading, or immediate provisionalization of the implants [27, 28]. Identification of the best treatment option is based on the implant stability at the time of installation, and various invasive and noninvasive techniques have been developed to determine this.

Histomorphometric evaluation and assessment of removal torque are the most frequently used invasive methods [29–31] as they provide reliable data regarding bone-implant contact, strength, and quality of implant anchorage. Microscopic observation of thin histological sections is the most widely used method of examining bone morphology and architecture. However, these destructive methods are only applicable to retrieval implants. Although this method provides high resolution images, it has several limitations (e.g., time consuming, requiring substantial preparation of the specimens including embedding in methyl methacrylate/paraffin followed by sectioning). Additionally, histomorphometry only allows two-dimensional (2D) evaluation of bone biomechanics and tissue healing, and clinical operations require nondestructive techniques for the evaluation of peri-implant conditions.

In recent years, resonance frequency analysis (RFA) and radiographic analysis microcomputed tomography (micro-CT) have been introduced as methods to measure implant stability [32, 33]. RFA uses the implant stability quotient (ISQ), which is a normalized dimensionless measurement detected using a commercial RFA device, to evaluate the status of implant-bone interfaces and determine dental implant stability based on bone-implant contact [34]. Increased bone-implant anchorage alters the resonance response due to changes in abutment-implant stiffness in the peri-implant bone [35]. The RF changes may indicate variation in bone-implant anchorage, thus giving an idea of implant stability in a noninvasive manner.

Micro-CT has been advocated as a technique to measure bone integration of dental implants and biomaterials [36, 37]. Histologic examination only provides limited 2D information, and the complex architecture of bone specimens can be better visualized using this technique. Previous studies have shown that the two-dimensional (2D) and three-dimensional (3D) parameters from micro-CT correlated significantly with those from conventional histomorphometric analyses [38], and the similarity between a micro-CT scan and histologic section was approximately 89% [39]. Thus, this nondestructive, fast, and precise technology allows 3D evaluation of bone biomechanics and hard tissue healing easily.

The aim of the present study was to use histologic evaluation, analysis of resonance frequency, and micro-CT to evaluate the response in the peri-implant bone of beagle dogs when placed in contact with two different surface-modified implants [GDP treatment (Ar-GDP) and fibronectin coating following GDP treatment (GDP-fib)] for 2–8 weeks.

## 2. Materials and Methods

### 2.1. Implant Preparation

**Figure 1:**
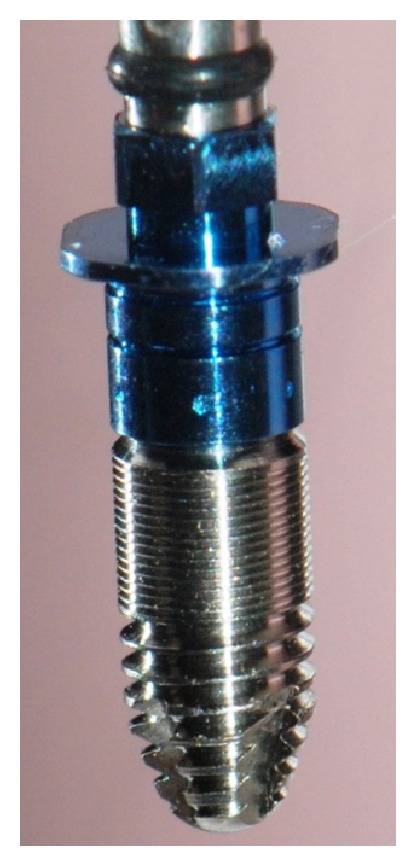
Experimental implant device: screw-shaped titanium implant packaged with fixture mount. The diameter and length of implant were 3.75 mm and 10 mm, respectively.

MG InHex (MOZO-GRAU®, Valladolid, Spain) implants made from commercially pure grade IV titanium and containing microthreads in the cortical part, self-tapping design in the apical end, and a 45° platform switching shoulder were used in the early healing study (Figure 1). The implants selected were 10 mm long and 3.75 mm in diameter.

**Figure 2:**
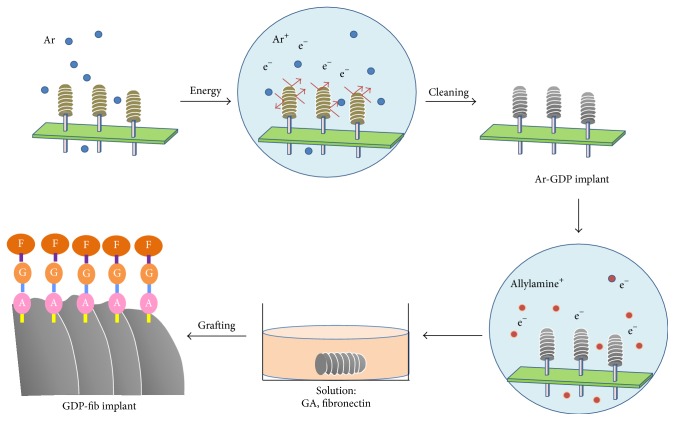
Schematic of sample preparation.

After sterilization with UV light overnight, the implant surfaces were treated with glow discharge plasma (GDP) and protein grafting, as described previously [40]. The implants were cleaned with argon-based GDP (PJ; AST Products Inc., North Billerica, MA, USA) at 85 Watts (W), 13.56 MHz, and 100 millitorr of argon gas at room temperature for 15 minutes (Figure 2). Implants treated with GDP only were used as control and have been defined as “Ar-GDP” in this paper.

Thereafter, the implants were exposed to allylamine gas in the GDP reactor for 30 minutes, immersed in 3% glutaraldehyde (GA) solution (Merck, NJ, USA) for 30 min, and rinsed with 0.1 M phosphate buffered saline (PBS). The implants were then immersed in 5 *μ*g/mL fibronectin solution (Sigma-Aldrich Co., St. Louis, MO, USA). Tris-phosphate buffer (pH 7.4) was used to interrupt the chain reaction of fibronectin links, and these implants were named “GDP-fib.”

### 2.2. Experimental Animals and Surgical Procedures

The study protocol was approved by the Animal Care and Ethics Committee of Taipei Level Biotechnology Inc., IACUC protocol #120301, Taipei, Taiwan. The study included six 6-month-old beagles, and all surgical procedures were performed under general anesthesia and with the supervision of veterinary surgeons.

**Figure 3:**
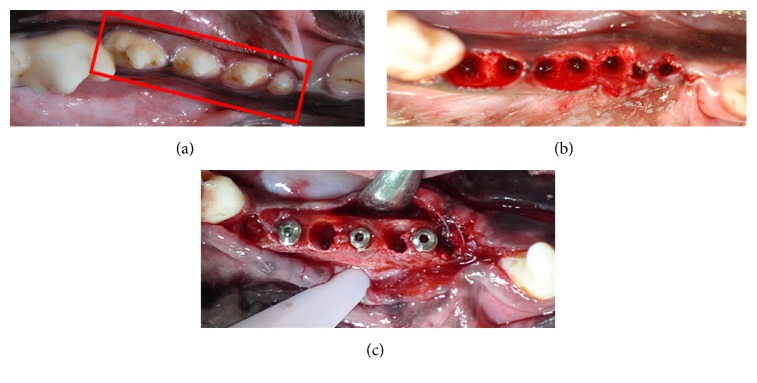
(a) Surgical area before extraction. First, second, third, and fourth premolars in the red box. (b) Surgical area after extraction. (c) Placement of implants.

The general anesthesia used included 0.1 mg/kg atropine, 10 mg/kg Zoletil 50 (Virbac Co., Carros, France), and 30 mg/kg Pentobarbital, and local infiltration anesthesia was performed with 2% lidocaine (3M-ESPE, Neuss, Germany) at the surgical site. All mandibular premolars were extracted, and the Ar-GDP and GDP-fib implants were placed in the fresh extraction holes (Figures 3(a), 3(b), and 3(c)). The implant site was prepared according to the recommendations of the manufacturer (MOZO-GRAU, Valladolid, Spain). A locator drill was used to first mark the implant sites, which were then drilled using 2.0, 3.0, and 3.3 conical drills at 1200 rpm along with profuse saline irrigation. Lastly, the countersink drill was used to prepare the cortical bone plate. A motor-driven hand piece with 35 Ncm and 20 rpm was used to carefully place the implants such that the body was completely buried in the bone, and the cover screw was at the alveolar crest level. Six implants (3 Ar-GDP implants as control and 3 GDP-fib implants) were installed blindly and randomly by the surgeon in the right and left mandibular premolar areas in each dog (three by side). Following implantation and RFA measurement, the buccal and lingual mucoperiosteal flaps were readapted such that the implants were submerged and sutured using interrupted absorbable sutures (Vicryl® 4.0, Ethicon, Somerville, NJ, USA). The animals were sacrificed, and the biopsies were collected after 2, 4, and 8 weeks to allow evaluation of the healing responses along the implants. The carcasses were stored in freezers and disposed by licensed contractors in accordance with the rules of LBAC (Level Biotechnology Animal Center).

### 2.3. Resonance Frequency Analysis (RFA)

Resonance frequency analysis (RFA) was performed to measure implant stability. Following implant insertion, the ISQ was measured in a buccolingual direction at each sacrificed interval using a commercial RFA device (Osstell Mentor®, Integration Diagnostics AB, Gothenburg, Sweden), and its values ranged from 0 to 100. For each implant, the measurement of ISQ value was repeated at least three times to verify accuracy, and the mean and standard deviation was used for statistical analysis.

### 2.4. Microcomputed Tomography (Micro-CT) Analysis

At 2, 4, and 8 weeks, the beagles were sacrificed using an overdose of Pentobarbital. Biopsies were collected at each interval to evaluate the healing around the implants. At the time of retrieval, the cover screws and the overlying soft tissue were removed. After measuring the ISQ value, the implant site was dissected into segments that contained one implant each using a trephine drill (6 mm in diameter). The individual implants with surrounding bone were fixed in 4% buffered formaldehyde solution immediately for micro-CT scanning and histological processing.

The cylindrical biopsies of bone blocks containing implants were analyzed by a micro-CT scanner, SkyScan 1076 desktop X-ray microtomography system (SkyScan, Kontich, Belgium), using an X-ray source set at 70 KV, 141 *μ*A with a 0.5 mm aluminum filter and 18.27 *μ*m image pixel size. Each 3D image data set contained approximately 800 micro-CT slice images with a resolution of 18 *μ*m. The *x*, *y*, and *z* axes of each specimen were corrected using DATAVIEWER v1.5.1 (SkyScan, Kontich, Belgium). Thereafter, a circular volume of interest (VOI) was measured on the implants using CTAn v1.14 (SkyScan, Kontich, Belgium). Starting at the first thread, a circular region of interest (ROI) was set closely along the implants to form the inner loop of the ROI. The radius of the outer loop was 0.5 mm more than the inner loop, and the ring-shaped VOI space equaled the outer loop minus the inner loop. Thereafter, the VOI was manually set to isolate the bone tissue, preserve bone structure, and exclude the implant material simultaneously.

The ratio of the bone volume (BV) to the total volume (TV) (BV/TV) was calculated. Binary selections of samples, made based on the grayscale density ranging between units of 30 and 120, were included in the calculation.

### 2.5. Histologic Evaluation

The fixed specimens were histologically evaluated following the micro-CT examination. The tissue blocks were dehydrated in a series of alcohol using standard histological techniques. The specimens were decalcified, and the implants were removed from bone blocks before being embedded with paraffin. Sections (2–5 *μ*m) of the embedded specimens were made by using a diamond-edged knife with hand-wheel (RM2235, Leica Biosystems Nussloch GmbH, Germany) in a direction parallel to the long axis of implants. The decalcified sections were then stained with H&E (hematoxylin and eosin stain). A digital histological scanning system (ScanScope®, Aperio CS scanner, Vista, CA, USA) was used to record images at 20x to 400x magnification. The resolution of the scanner was 0.5 *μ*m/0.25 pixels, and the images were digitized with the help of an image analysis software (ImageScope®, Vista, CA, USA).

### 2.6. Statistical Analysis

The mean values and standard deviations (SD) of the RFA and micro-CT analysis were calculated. The data were compared using Student's *t*-test, and statistical significance was set at *P* < 0.05. The relationship between the ISQ values and BV/TV ratio from the micro-CT were analyzed using a regression line. Regression parameters were also calculated to describe the linear relation between RFA and micro-CT measurements. All statistical analyses were performed on Excel 2010 for Windows.

## 3. Results

All six beagles were healthy throughout the experimental period. The gingival tissues surrounding the healing abutment did not show any postoperative wound healing complications, and the oral mucosa surrounding the dental implants did not show any remarkable inflammatory symptoms. Moreover, the dogs did not show any remarkable changes in their body weights throughout the study.

### 3.1. Implant Stability Analysis

**Figure 4:**
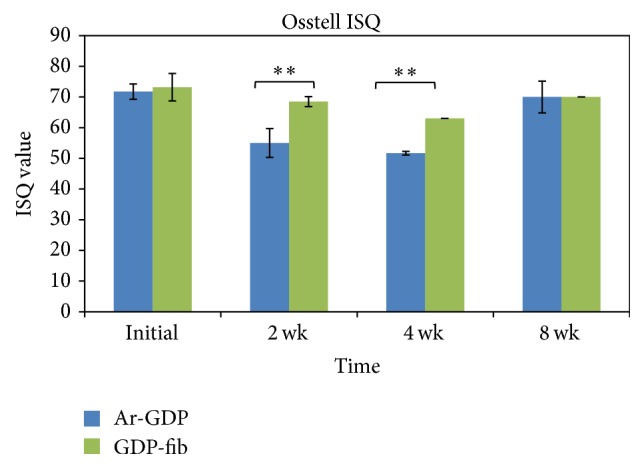
Mean implant stability (implant stability quotient (ISQ) ± SD) of Ar-GDP and GDP-fib implants 2, 4, and 8 weeks after placement. The stability of Ar-GDP implants was significantly lower at 2 and 4 weeks compared to GDP-fib implants (^*∗∗*^ *P* < 0.01).

At the time of implantation, the mean ISQ values of Ar-GDP and GDP-fib implants were both >70 (71.77 and 73.17 separately). These values decreased at 2 and 4 weeks but returned to 70 at 8 weeks (Figure 4). The ISQ values of GDP-fib implants were significantly greater than the Ar-GDP implants at 2 and 4 weeks (*P* < 0.01). At 8 weeks after implantation, the ISQ mean values showed no statistically significant differences between the Ar-GDP and the GAP-fib implants. Nevertheless, the mean ISQ values of GDP-fib implants were equal to or greater than the Ar-GDP implants during the study period.

### 3.2. Micro-CT Analysis

**Figure 5:**
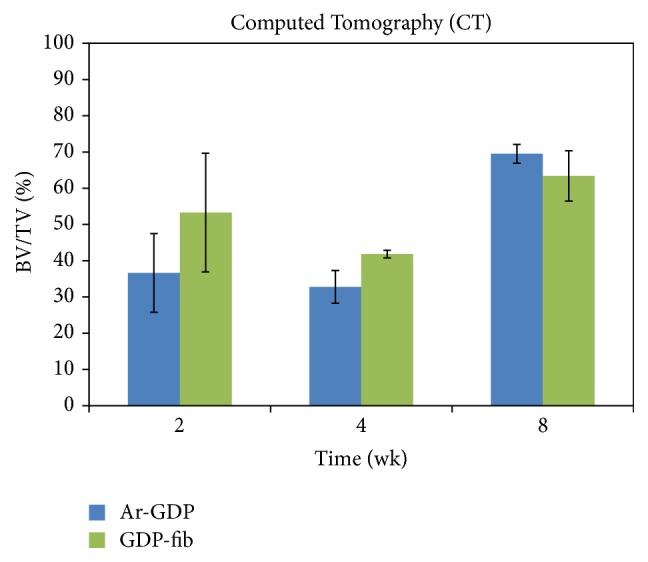
Mean values of bone-implant contact calculated by micro-CT analysis.

**Figure 6:**
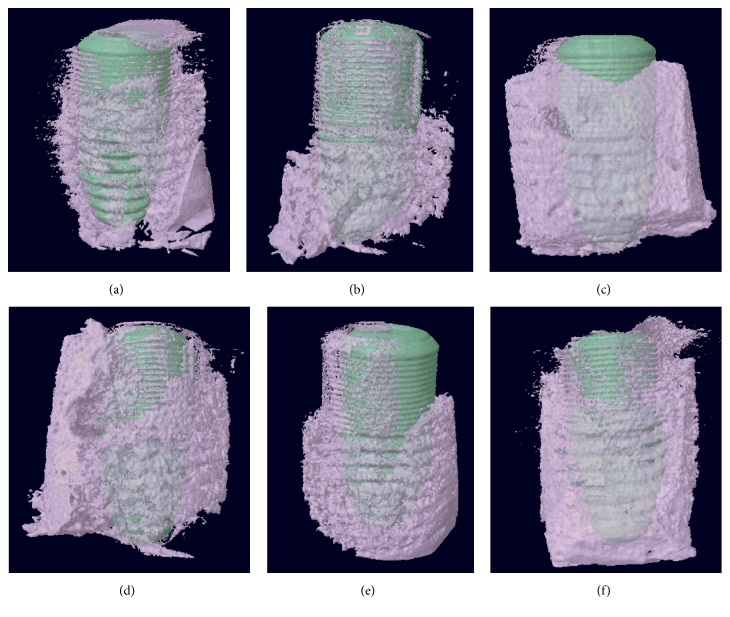
Schematic of Ar-GDP/GDP-fib implants with surrounding bone at 2, 4, and 8 weeks after implantation. (a) Ar-GDP implant at 2 weeks, (b) Ar-GDP implant at 4 weeks, (c) Ar-GDP implant at 8 weeks, (d) GDP-fib implant at 2 weeks, (e) GDP-fib implant at 4 weeks, and (f) GDP-fib implant at 8 weeks.

The BV/TV ratio was calculated as a percentage ratio for each specimen. At 2 and 4 weeks, the BV/TV ratio of GDP-fib implants was greater than that of Ar-GDP implants (Figure 5), suggesting that there was greater new bone formation on the fibronectin coated surfaces. There was a tendency for a decrease in growth between 2 and 4 weeks, but this rose again after 8 weeks. The decreased BV/TV ratio indicates a loss of mechanical stability during the initial healing stage. No significant differences were observed between the two groups at each interval.  Figure 6 shows real images of separate Ar-GDP/GDP-fib implants scanned by micro-CT, and these could be used to examine the bone surrounding each group of implants. At 2 weeks, the bone surrounding GDP-fib implants was greater than the Ar-GDP implants. However, the surrounding bone decreased at 4 weeks in both groups and was seen to mature after 8 weeks of healing.

### 3.3. Descriptive Histological Analysis

**Figure 7:**
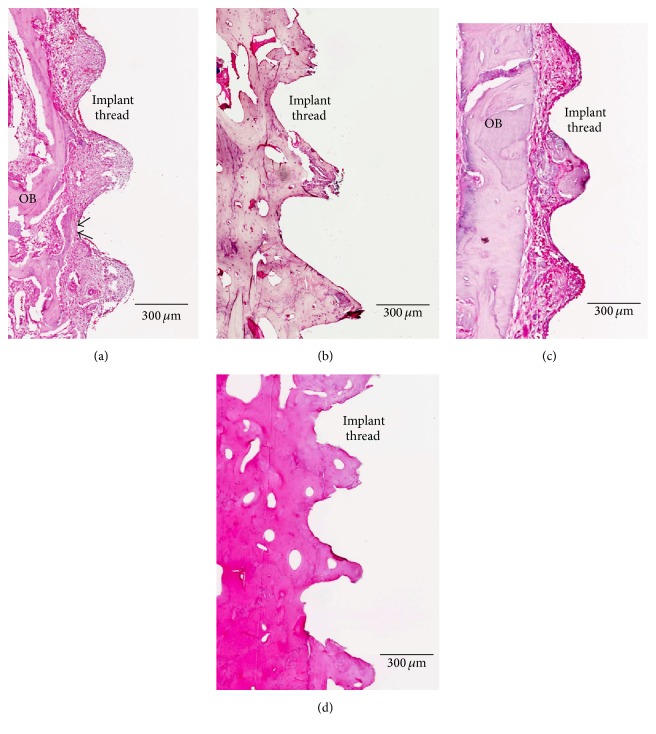
Ground sections of the peri-implant tissue in the GDP-fib and Ar-GDP groups after 2 and 8 weeks of healing. (a) Wound chamber of the GDP-fib implant at 2 weeks, decalcified section, original mag. ×80. (b) Wound chamber of the GDP-fib implant at 8 weeks, decalcified section, original mag. ×80. (c) Wound chamber of the Ar-GDP implant at 2 weeks, decalcified section, original mag. ×80. (d) Wound chamber of the Ar-GDP device at 8 weeks, decalcified section, original mag. ×80.

Figure 7 shows a cross-section (ground section) of Ar-GDP and GDP-fib implants with surrounding soft and hard tissue from biopsies sampled 2 and 8 weeks after implantation. At the 2nd week, compact parent bone (OB) and provisional matrix were seen in both GDP-fib (Figure 7(a)) and Ar-GDP (Figure 7(c)) groups. The majority of the space between the old bone (OB) and the implant surface was filled with soft tissue containing abundant blood vessels and bone debris particles. Early bone apposition occurred especially at sites where OB was in contact with or very close to the implant surface (black arrows, Figure 7(a)). After 8 weeks of healing, lamellar bone with parallel fibers was evident adjacent to the bone-implant surfaces, indicating advanced stage of bone maturation. This lamellar bone tissue represents mature bone in direct contact with the implant threads, and the minimal presence of osteoblasts and inflammatory cells suggests that bone remodeling was still occurring at 8 weeks (Figures 7(b) and 7(d)).

**Figure 8:**
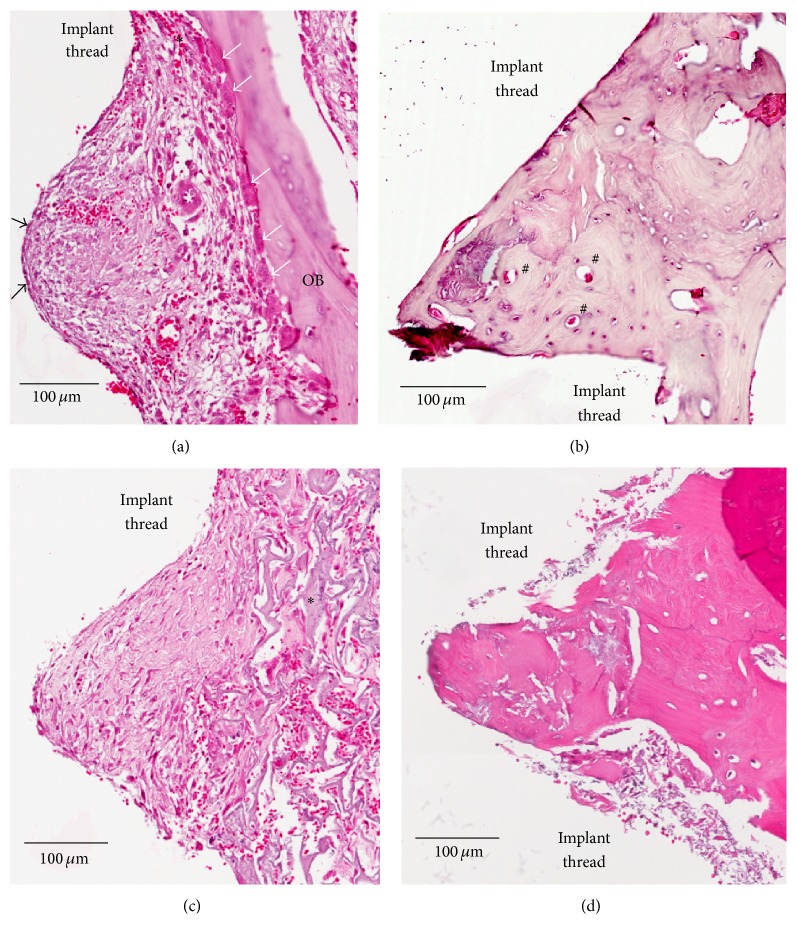
Ground sections of the peri-implant tissue of GDP-fib and Ar-GDP implant surfaces after 2 and 8 weeks of healing. (a) Wound chamber of the GDP-fib implant at 2 weeks. Osteoclasts (*white arrows*) and osteoblasts (*black arrows*) were surrounded by provisional matrix, decalcified section, original mag. ×200. (b) Wound chamber of the GDP-fib implant at 8 weeks. Osteon (#) could be clearly identified, decalcified section, original mag. ×200. (c) Wound chamber of the Ar-GDP implant at 2 weeks. Woven bone (*∗*) formation extending into provisional connective tissue matrix was seen, decalcified section, original mag. ×200. (d) Wound chamber of the Ar-GDP implant at 8 weeks, decalcified section, original mag. ×200.

The experimental chamber unit has been defined as the peri-implant tissue between two implant threads in this paper.  Figure 8 provides a closer view of the quality of bone response surrounding implants.  Figure 8(a) illustrates the experimental chamber units of the GDP-fib implants after 2 weeks of healing. Bone remodeling was observed on the parent bone surface and extending partly into the implant surface (*∗*) in the transitional region between the compact parent bone (OB) and implant surface. Osteoblasts (*black arrows*) and provisional matrix abundant in spindle-shaped cells, collagen fibers, vascular units, and tiny trabeculae of woven bone were seen to line the implant surface. Multinuclear osteoclasts (*white arrows*) could also be seen on the surface of old parent bone (OB). After 8 weeks of healing (Figure 8(b)), a majority of the wound chamber appeared to be occupied by bone. The formation of osteons (#) and lamellar bone with parallel fibers indicates an advanced stage of bone maturation. Figures 8(c) and 8(d) show the peri-implant tissue of Ar-GDP implants after 2 and 8 weeks of healing. Moreover, woven bone formation (*∗*) extending into a provisional connective tissue matrix could also be seen after 2 weeks of healing. Lamellar bone with parallel fibers could also be seen on the surface of Ar-GDP implants at the 8-week interval. Osteoblasts, osteocytes, and inflammatory cells were present in the experimental chamber unit.

### 3.4. Correlations

**Figure 9:**
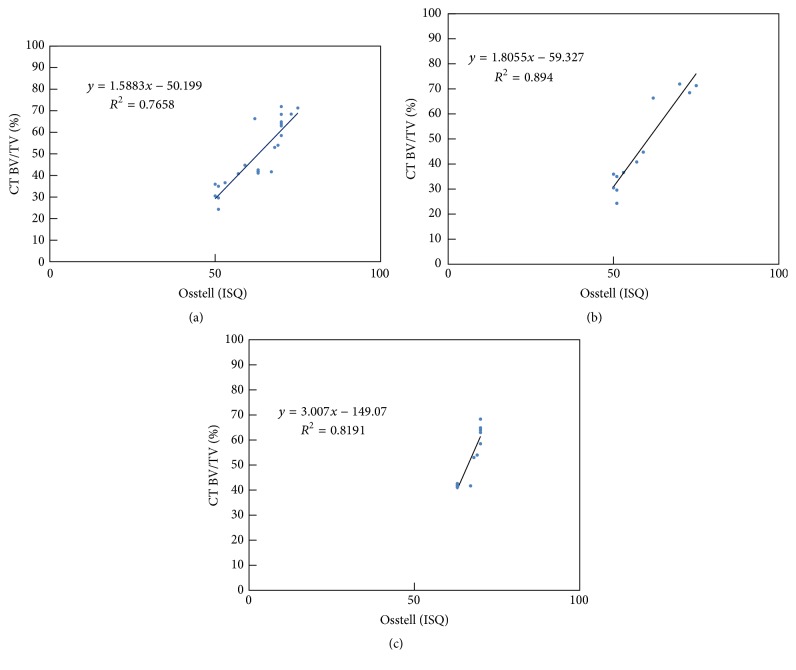
Relationship of the 3D bone-implant contact (BIC) measurements between the BV/TV ratio from CT analysis and the RF data in (a) all, (b) Ar-GDP, and (c) GDP-fib implants.

Replotting the measurements in RA analysis (Figure 4) and micro-CT analysis (Figure 5) in Figure 9(a), a linear correlation was obtained between the ISQ values and BV/TV ratio from micro-CT measurements (*R* ^2^ = 0.7658, *P* < 0.01). The linear regression coefficient (*R* ^2^) between RFA and micro-CT measurements was 0.894 (*P* < 0.05) in the Ar-GDP group and 0.8191 (*P* < 0.01) in the GDP-fib group.

## 4. Discussion

The main objective of this investigation was to compare the effects of surface modification with either GDP treatment or fibronectin coating following GDP treatment (GDP-fib) on the peri-implant bone response. These implants were placed in fresh extraction holes in the mandibular premolar area of beagles in order to mimic the clinical situation immediately after implantation. To minimize differences in bone density and variations between animals, the test and control implants were randomly placed in the same animal.

Previous studies examining the characteristics of modified surfaces [23, 24] found that the surface roughness, surface wettability, and cell viability of titanium disks were enhanced with fibronectin coating. FITC labeling was used to confirm the presence of fibronectin coating on the titanium surfaces. Hydrophilic titanium surfaces have been reported to play an important role in the differentiation and growth factor production of osteogenic cells [41]. In general, implants with higher surface roughness exhibit greater bone-implant contact [7, 8, 30]. It has also been demonstrated that modification of the implant surface by coating it with fibronectin roughens its topography, enhances healing responses, and shows faster directed bone formation [26, 42, 43].

There are several methods of evaluating implant stability, of which resonance frequency analysis (RFA) allows noninvasive and nondestructive quantitative measurement. The output data from the commercial RFA devices is known as the implant stability quotient (ISQ). RF is altered when there is increased bone-implant anchorage due to changes in the interface between implants and peri-implant bone [35]. Its efficacy in determining the healing status and implant stability has been confirmed by using* in vivo* analysis to compare it to histological analysis [4]. With the development of technology, the 3D structures of bone biopsies can now be evaluated using micro-CT. This fast and precise technique allows qualitative and quantitative measurements of bone-implant integration and also allows 3D evaluation of the complex architecture of bone.

Analysis of RF data showed that there were no significant differences between the Ar-GDP and GDP-fib groups at the time of implantation (71.77 ± 2.49 and 73.17 ± 4.49 separately). At 2 and 4 weeks after implantation, ISQ values decreased in both groups, and this was in agreement with earlier studies [29, 32]. This suggests a loss of mechanical stability with increased pressure from implant threads during the initial healing stage. This phenomenon was also apparent in the micro-CT results. The surrounding bone at 4 weeks was less than that at 2 weeks in both groups, although this difference was not statistically significant. The ISQ values increased and returned to a value of 70 (i.e., the overall average ISQ value of all implants over time) in both groups after 8 weeks of healing. The observed changes in ISQ values were most likely a result of the apposition of woven bone and parallel-fiber bundle bone on the implant surfaces. Additionally, the mean ISQ values of GDP-fib implants were equal to or greater than that of Ar-GDP implants at every interval. Statistically significant differences were observed between these two groups at 2 and 4 weeks after surgery.

Previous studies have proposed that implants are capable of immediate loading when the ISQ value is >60, and values <40 should be considered as a warning of early failure of implants [5]. The ISQ values of the GDP-fib implants were >60 and those of the Ar-GDP implants were >50 throughout the study period. The relationship between RFA and micro-CT measurements was linear, with “*R*” being >0.9 in both groups. This suggests a high positive correlation between RFA and micro-CT measurements. Significant differences were observed in correlations (replotting measurements in ISQ values and BV/TV ratio) of Ar-GDP implants (*P* < 0.05) and GDP-fib implants (*P* < 0.01). The histologic findings demonstrated a change of peri-implant tissue from provisional matrix to parallel-fibered and lamellar bone. The presence of osteons indicated an advanced stage of bone maturation around the implants. Nevertheless, both RFA and micro-CT revealed the 3D architecture of the bone surrounding the implants, while histologic ground sections provided a 2D planar view only.

## 5. Conclusion

Based on the results of this study, it can be concluded that biological modification of implants enhances its stability. Moreover, coating with fibronectin may offer some advantages, such as shortened treatment time, immediate loading, or immediate provisionalization of the implants. However, the number of samples available was limited, preventing inclusion of unmodified implants. Further studies examining the histomorphometry of noncoated implants are necessary. The results of this study may serve as a useful reference for further* in vivo* studies.
